# Cr-resistant rhizo- and endophytic bacteria associated with *Prosopis juliflora* and their potential as phytoremediation enhancing agents in metal-degraded soils

**DOI:** 10.3389/fpls.2014.00755

**Published:** 2015-01-06

**Authors:** Muhammad U. Khan, Angela Sessitsch, Muhammad Harris, Kaneez Fatima, Asma Imran, Muhammad Arslan, Ghulam Shabir, Qaiser M. Khan, Muhammad Afzal

**Affiliations:** ^1^Environmental Biotechnology Division, National Institute for Biotechnology and Genetic EngineeringFaisalabad, Pakistan; ^2^Bioresources Unit, Austrian Institute of Technology GmbHTulln, Austria; ^3^Earth Sciences Department, King Fahd University of Petroleum and MineralsDhahran, Saudi Arabia

**Keywords:** *Prosopis juliflora*, heavy metals, phytoremediation, Cr-resistant bacteria, plant growth-promoting bacteria, rhizobacteria, endophytic bacteria

## Abstract

*Prosopis juliflora* is characterized by distinct and profuse growth even in nutritionally poor soil and environmentally stressed conditions and is believed to harbor some novel heavy metal-resistant bacteria in the rhizosphere and endosphere. This study was performed to isolate and characterize Cr-resistant bacteria from the rhizosphere and endosphere of *P. juliflora* growing on the tannery effluent contaminated soil. A total of 5 and 21 bacterial strains were isolated from the rhizosphere and endosphere, respectively, and were shown to tolerate Cr up to 3000 mg l^−1^. These isolates also exhibited tolerance to other toxic heavy metals such as, Cd, Cu, Pb, and Zn, and high concentration (174 g l^−1^) of NaCl. Moreover, most of the isolated bacterial strains showed one or more plant growth-promoting activities. The phylogenetic analysis of the 16S rRNA gene showed that the predominant species included *Bacillus*, *Staphylococcus* and *Aerococcus*. As far as we know, this is the first report analyzing rhizo- and endophytic bacterial communities associated with *P. juliflora* growing on the tannery effluent contaminated soil. The inoculation of three isolates to ryegrass (*Lolium multiflorum* L.) improved plant growth and heavy metal removal from the tannery effluent contaminated soil suggesting that these bacteria could enhance the establishment of the plant in contaminated soil and also improve the efficiency of phytoremediation of heavy metal-degraded soils.

## Introduction

Soil contamination by chromium and other toxic heavy metals has been a major problem worldwide. Among other industries, tanneries belong to the main contributors of soil and water contamination with Cr and other toxic heavy metals (Tariq et al., 2008; Rajkumar et al., 2012; Reichman, 2014). The presence of Cr and other toxic heavy metals in the environment could be highly toxic to human health (Chen et al., 2010; Ma et al., 2011; Sagar et al., 2012; Gil-Cardeza et al., 2014).

A number of woody plant species can grow on heavy metal polluted soil and are known as indicators of heavy metal pollution in the soil (Capuana, 2011). *Prosopis juliflora*, (Sw.) DC, a multipurpose perennial tree native to South America (Sajjad et al., 2012), was also studied as a possible bioindicator of soil pollution (Senthilkumar et al., 2005). In many parts of the world it is a well-known plant species for its use as a fuel, shade, timber and forage. It is a deep rooted bush or tree and widely propagated in Asia, particularly in India and Pakistan (Deans et al., 2003; Benata et al., 2008; Qureshi et al., 2014). Furthermore, it remediates soil contaminated with heavy metals and helps in site reclamation (Jamal, 2006; Usha et al., 2009; Varun et al., 2011). During an initial survey of the tannery effluent contaminated area of Kasur (Punjab, Pakistan), which is one of the most polluted areas by heavy metals in the world, only *P. juliflora* has been found to grow on the contaminated area with heavy metal high concentrations (Cd, 26 mg kg^−1^; Co 22 mg kg^−1^; Cr, 2243 mg kg^−1^; Fe, 137 mg kg^−1^; Mn 9.4 mg kg^−1^; Ni, 34 mg kg^−1^; Pb, 18 mg kg^−1^; Zn, 14 mg kg^−1^) (Afzal et al., 2014a). Thus, there is a need for the soil in that area to be remediated and to make it usable again.

The combined use of plants and heavy metal-resistant plant growth-promoting bacteria is a promising approach for the remediation of heavy metal contaminated soil (Ma et al., 2009; Rajkumar et al., 2012; Sessitsch et al., 2013; Reichman, 2014). Rhizobacteria colonize in the close vicinity of roots whereas endophytic reside within the plant tissues (Afzal et al., 2014b). These bacteria may reduce the toxicity of heavy metals in soil and plant due to their metal-resistance and bioaccumulation potential (Gadd, 2010; Sessitsch et al., 2013; Zhu et al., 2014). Moreover, they may improve plant growth and development in a contaminated soil due to different plant growth-promoting activities (Glick, 2010; Sessitsch et al., 2013; Andrades-Moreno et al., 2014).

Better knowledge of the type of bacteria colonizing the rhizosphere and endosphere of the plants growing on heavy metal contaminated soil is important, however, rhizosphere and endophytic bacterial communities associated with *P. juliflora* have not been investigated so far. The aim of this study was to (i) explore the type of Cr-resistant rhizosphere and endophytic bacteria associated with *P. juliflora*, growing on the tannery effluent contaminated soil, (ii) and to study the effect of inoculation of three isolates to enhance plant growth and accumulation of heavy metals in the root and shoot of ryegrass vegetated in the tannery effluent contaminated soil.

## Materials and methods

### Plant material and soil sampling

Plants of *P. juliflora* were collected in July 2013 from the site located in the surrounding of tanneries of Kasur (31°0.7′ N. 74°0.27′ E). Rhizosphere soil was collected from three different plants. Plants were carefully dug out with an intact root system and the soil tightly adhering to the roots was collected. The rhizosphere soil was obtained by agitating roots and sampling the soil still attach to the roots. No vegetation was observed in the bulk soil. Bulk soil samples were collected from three different points which were 100 ft away from the vegetation. The shoots of three *P. juliflora* plants were cut from the roots at the collar diameter.

### Isolation and characterization of cr-resistant RHIZO- and endophytic bacteria

Cr-resistance endophytic bacteria were isolated from the root and shoot of *P. juliflora* as described earlier (Zhu et al., 2014). Briefly, the roots and shoots were carefully washed and surface sterilized with 70% ethanol and 1% bleach. Subsequently, 3 g surface sterilized shoots or roots were homogenized with a pestle and mortar in 10 ml NaCl (0.9%, w/v) solution. The homogenized material was agitated for 1 h at 30°C. After settling of solid material, serial dilutions up to 10^−2^ were plated onto solid LB medium containing 100 mg l^−1^ Cr as Cr_2_(SO_4_)_3_. In an earlier study, a relatively low number of rhizosphere bacteria was obtained due to the presence of Cr above 100 mg l^−1^ (Abou-Shanab et al., 2005), therefore, in this study, 100 mg l^−1^ Cr concentration was used to obtain maximum number of Cr-resistant bacteria. Several studies showed that metals influence microorganisms by adversely affecting their growth, morphology and biochemical activities, resulting in a decrease in their biomass and numbers (Giller et al., 1998; Abou-Shanab et al., 2005). Cr-resistant rhizosphere bacteria were obtained as described earlier (Kuffner et al., 2008). The soil slurry was prepared by mixing 4 g soil with 12 ml of 0.9% NaCl solution, agitated for 1 h at 30°C. After the settlement of soil particles, serial dilutions up to 10^−3^ were plated onto LB media containing Cr 100 mg l^−1^. Colonies with different morphologies were picked out and purified by re-streaking onto the same medium at least three times.

On the basis of cell morphology, 78 different morphotypes were identified. A restriction fragment length polymorphism (RFLP) analysis of the 16S–23S rRNA intergenic spacer (IGS) region was performed to distinguish these 78 different bacterial morphotypes (Rasche et al., 2006; Yousaf et al., 2010a). On the basis of RFLP analysis, 26 different patterns (IGS-type) were obtained (Mastretta et al., 2009). A representative isolate of each IGS type was identified by partial 16S rRNA gene sequencing. 16S rRNA genes were amplified by using PCR primers 8f (5′-AGAGTTTGATCCTGGCTCAG-3′) and 1520 rev (5′-AAGGAGGTGATCCAGCCGGA-3′) as explained earlier (Rasche et al., 2006; Yousaf et al., 2010b). The PCR amplification products were sequenced by the Macrogen (Seoul, Korea) with 8f and 1520 rev primers. The sequences were compared with sequences in the GenBank database using NCBI Blast program (http://blast.ncbi.nlm.nih.gov/Blast.cgi). Sequences were submitted to GenBank database under accession numbers KJ933397-KJ933406, KJ999602-KJ999614, KM067905-KM067907. In addition, the isolated rhizosphere and endophytic bacterial strains were deposited in the NIBGE Biological Resource Centre (NBRC).

### Heavy metal analysis of soil and plant samples

For soil analysis, the samples (in three independent replicates) were air-dried, sieved, and 0.3 g soil digested with 1:1 concentrated HNO_3_–H_2_SO_4_. After cooling, the volume was made up to 100 ml with double de-ionized water. For plant analysis, separately, 10 g dried roots and shoots were ground to pass through 0.2 mm sieve and digested (1 g of each) with mixture of sulfuric acid (H_2_SO_4_), nitric acid (HNO_3_) and perchloric acid (HClO_4_) (Afzal et al., 2014c). The digested soil and plant samples were analyzed by inductively coupled plasma optical mission spectrometry (ICP-OES) for different heavy metals.

### Cr-resistance by the RHIZO- and endophytic bacteria

To determine the Cr-resistance of the isolated rhizosphere and endophytic bacteria, 100 μl of overnight grown cultures were streaked on LB agar media containing Cr 500, 1000, 2000, and 3000 mg L^−1^ as Cr_2_(SO_4_)_3_. In this study, the concentration of Cr in the rhizosphere and bulk soil was 2542 and 2243 mg kg^−1^, respectively, therefore, isolated bacterial strains were tested at different concentrations of Cr up to 3000 mg l^−1^ for their possible application in the phytoremediation of tannery effluent contaminated soil. All the plates were incubated at 37°C for 2 days and observed for the appearance of bacterial growth. The resistance was expressed as the maximum tolerable concentration of Cr, which is defined as maximum concentration of Cr not effecting bacterial growth.

### Tolerance to other heavy metals and NaCl

All the isolated Cr-resistant bacteria were also exposed to different heavy metals (Cd, Cu, Pb, and Zn) and NaCl to determine their tolerance to the heavy metals and NaCl as described earlier (Sagar et al., 2012). Briefly, all the isolated rhizosphere and endophytic bacteria were streaked on LB agar media supplemented with different metals at different concentrations: CdCl_2_ (100 mg l^−1^), CuCl_2_(100 mg l^−1^), PbNO_3_(100 mg l^−1^), ZnSO_4_(100 mg l^−1^) and NaCl (1 M, 2 M, 3 M, and 3.5 M). As the concentration of most of the heavy metals in the tannery effluent contaminated soil is below 100 mg kg^−1^, therefore, isolated bacterial strains were tested at 100 mg kg^−1^ of the heavy metals for their possible application in bacterial assisted phytoremediation of tannery effluent contaminated soil.

### Determination of plant growth-promoting properties of the isolated bacteria

Different plant growth-promoting activities, such as 1-aminocyclopropane-1-carboxylate (ACC) deaminase, siderophore and indole acetic acid (IAA) production and solubilize phosphorous were determined in all the isolated bacteria using the protocols as described earlier (Naveed et al., 2014). Briefly, ACC deaminase activity of the isolates was tested on minimal medium containing 0.7 g ACC L^−1^ as sole nitrogen source. Phosphate solubilization activity was determined by the formation of clear zone around bacterial growth on Pikovskaya's agar medium. Bacterial isolates were assayed for siderophore production on the Chrome azurol S (CAS) agar medium. The IAA production activity was determined using Salkowski reagent.

### Effect of inoculation of bacteria on plant growth

Three different endophytic bacterial strains (*Pantoea stewartii* strain ASI11, *Microbacterium arborescens* strain HU33 and *Enterobacter* sp. strain HU38) were grown in LB broth overnight and cells were recovered by centrifugation and re-suspended in 0.9% (w/v) NaCl solution. These bacteria exhibited Cr-resistance as well as plant growth-promoting activities. The compatibility of these three strains was tested by cultivating together on LB medium for 24 h and then plating serial dilutions of the culture on LB plates. Three different colonies, corresponding to ASI11, HU33, and HU38, could be isolated from LB plates, showing compatibility between the selected strains (data not shown). Ryegrass was shown to tolerate Cr and other heavy metals contamination in previous experiments (Duquène et al., 2009; Chigbo and Batty, 2013; Lou et al., 2013) and was therefore chosen as experimental plant. Surface sterilized seeds (200) of ryegrass were sown in the tannery effluent contaminated soil (Cr content, 2243 mg kg^−1^; pH 7.11; Na 10370 mg kg^−1^; Cl 4410 mg kg^−1^; SO_4_ 1081 mg kg^−1^; PO_4_ 30 mg kg^−1^; NO_3_ 657 mg kg^−1^) in plastic pots (1.5 kg soil pot^−1^) and bacterial inoculum was applied individually as well as in combination over the soil surface immediately after sowing the seeds as described earlier (Afzal et al., 2013). Before sowing, the soil was treated with 50 ml inoculant suspension (app. 10^10^ cfu/ml) containing mixture of ASI11, HU33, and HU38 or sterile 0.9% NaCl solution. The combined inoculum containing equal numbers of each strain. Our previous experiments showed that inoculum density affects bacterial survival, colonization and phytoremediation efficacy, and maximum phytoremediation achieved at high inoculum density. Therefore, in this study, high density inoculum (10^10^ cfu/ml) was used instead low inoculum density (10^7^–10^8^ cfu/ml). Treatment without bacterial inoculation was set as control. The pots were put under ambient conditions of temperature and light (1st March 2014–30th May 2014) in the vicinity of National Institute for Biotechnology and Genetic Engineering (NIBGE), Faisalabad, Pakistan. Percentage seed germination was determined after 1 week of sowing. After 3 months, plants were harvested, root and shoot length and dry weight were determined. Bacterial population sizes in the rhizosphere and endosphere of ryegrass were determined by plate count method on LB medium containing 500 mg L^−1^ Cr as Cr_2_(SO_4_)_3_ as described earlier (Afzal et al., 2012). Thirty colonies of each treatment were randomly picked and the identity of isolates with the inoculant strain was confirmed by restriction fragment length polymorphism (RFLP) analysis of the 16S–23S rRNA intergenic spacer region (IGS) (Andria et al., 2009). Isolates and inoculant strains had identical restriction patterns. Bulk soil, root and shoot samples were analyzed for Cr and other heavy metals as described earlier (Afzal et al., 2014c).

### Statistical analysis

SPSS software package version 17.0 (SPSS, Inc., Chicago, IL) was used for analyzing data for seed germination, shoot and root length and weight. The data (three replicates of each treatment) were subjected to analysis of variance (ANOVA), and the means [±standard deviation (SD)] were compared using Duncan's multiple range test. Soil heavy metal concentrations were compared with One-Way of ANOVA. Plant heavy metal concentrations were analyzed by a paired *t*-test using Statistix Version 8.1; Statistix, Tallahasee, Florida, USA. Microbial enumeration data were subjected to Two-Way ANOVA. Mean separation was done using LSD at *p* = 0.05.

## Results

### Heavy metals contents in soil and plant

Heavy metal concentrations were significantly higher (*p* = 0.0309 and 0.0280, respectively) in the rhizosphere than in roots and shoots (Table 1). Similarly, heavy metal concentrations were significantly higher (*p* = 0.0096) in roots as compared to shoots. Based on mean values, heavy metals in the rhizospheric soil of *P. juliflora* follow the declining concentration (mg kg^−1^) order: Cr (2542)>Fe (154)>Cu (72)>Cd (37)>Ni (30)>Co (28)>Pb (22)>Zn (17)>Mn (13).

**Table 1:** **The concentration of different heavy metals present in the bulk soil, rhizosphere, root and shoot of *Prosopis juliflora* growing on the tannery effluent contaminated soil**.

	**Cd mg kg^−1^**	**Co mg kg^−1^**	**Cr mg kg^−1^**	**Cu mg kg^−1^**	**Fe mg kg^−1^**	**Mn mg kg^−1^**	**Ni mg kg^−1^**	**Pb mg kg^−1^**	**Zn mg kg^−1^**
Bulk soil	26 (2.6)	22 (1.9)	2243 (165)	56 (4.6)	137 (12)	9.4 (1.8)	34 (5.8)	18 (2.5)	14 (1.6)
Rhizosphere	37 (3.3)	28 (4.4)	2542 (136)	72 (5.3)	154 (10)	13 (1.4)	30 (4.7)	22 (3.8)	17 (1.8)
Root	19 (2.1)	16 (2.3)	427 (28)	24 (2.7)	104 (7.8)	9 (1.7)	16 (2.1)	14 (1.4)	14 (2.2)
Shoot	15 (1.8)	12 (1.9)	284 (37)	19 (1.4)	58 (5.1)	7 (0.6)	13 (1.5)	12 (1.1)	12 (1.4)

*Each value is the mean of three replicates, the standard error of three replicates is presented in parentheses*.

### Culturable bacteria in the rhizosphere and endosphere of *P. juliflora*

Among all the isolates on the LB plates, 78 colonies were chosen according to their morphological differences and were differentiated into 26 groups according to their 16S–23S rRNA IGS RFLP patterns. A representative isolate of each IGS type was identified by partial 16S rRNA gene sequencing. The 26 isolates belonged to different genera and the predominant genera included *Bacillus*, *Staphylococcus* and *Aerococcus* (Table 2). A higher number of genera were obtained from the endosphere than the rhizosphere, and among the endophytic bacteria 95% were isolated from the shoot interior, whereas only 5% were obtained from the root interior.

**Table 2:** **The diversity of bacteria isolated from the rhizosphere (RH), root interior (RI) and shoot interior (SI) of *Prosopis juliflora* growing on the tannery effluent contaminated soil**.

**Strain name**	**Plant compartment**	**NCBI accession number**	**Most closely related species (sequence similarity, %)**	**Length (bp) of 16S rRNA gene sequenced**
PJSI1	SI	KJ999602	*Staphylococcus saprophyticus* (99)	1445
PJSI12	SI	KJ999603	*Massilia*sp. (99)	1133
PJSI13	SI	KJ999604	*Ochrobactrumintermedium* (99)	1351
PJRS17	RH	KJ999605	*Arthrobacter* sp. (99)	1404
PJRS20	RH	KJ999614	*Pseudomonas aeruginosa* (99)	1426
PJRS25	RI	KJ999606	*Bacillus* sp. (99)	1122
PJRI21	RH	KM067905	*Bacillus licheniformis*(99)	776
PJSI41	SI	KJ999610	*Bacillus pumilus* (99)	820
PJSI46	SI	KJ999611	*Staphylococcus* sp. (100)	1102
PJSI34	SI	KJ999607	*Aerococcus* sp. (99)	1128
PJSI9	SI	KJ999613	*Staphylococcus epidermidis* (99)	1136
UK09	SI	KM067907	*Staphylococcus epidermidis* (99)	1093
ASI	SI	KJ933397	*Staphylococcus epidermidis* (99)	1110
PJSI36	SI	KJ999608	*Aerococcus* sp. (99)	1431
ASI11	SI	KJ933399	*Pantoea stewartii* (99)	782
ASI14	SI	KJ933400	*Ochrobactrum* sp. (99)	1094
SISI43	SI	KJ933406	*Bacillus aerophilus* (100)	1434
PJRI24	RI	KM067906	*Staphylococcus* sp. (99)	926
RSA27	RH	KJ933401	*Bacillus licheniformis* (99)	1105
HU33	SI	KJ933403	*Microbacteriumarborescens* (99)	1082
PASI10	SI	KJ933398	*Aerococcusviridans* (100)	1122
PJSI37	SI	KJ999609	*Brevundimonasvesicularis* (99)	1111
HU38	SI	KJ933404	*Enterobacter* sp. (99)	1426
SISI39	SI	KJ933405	*Bacillus aquimaris* (99)	1440
PJRS31	SI	KJ999612	*Pseudomonas* sp. (99)	1427
RSAUK31	RH	KJ933402	*Pseudomonas stutzeri* (99)	1104

### Cr-resistance of the isolated bacterial strains

All isolated rhizosphere and endophytic bacteria were able to grow at concentration 500 mg L^−1^ Cr and could be considered resistant to this metal. It is important to note that only four bacteria (*Pseudomonas aeruginosa* sp. strain PJRS20, *Pantoea stewartii* sp. strain ASI11, *Microbacterium arborescens* sp. strain HU33 and *Enterobacter* sp. strain HU38) were able to grow at higher concentration of Cr (3000 mg L^−1^). The maximum tolerable concentration of Cr for each isolate is shown in Table 3.

**Table 3:** **Heavy metal and NaCl tolerance of the bacteria isolated from the rhizosphere and endosphere of *Prosopis juliflora* growing on tannery effluent contaminated soil**.

**Bacterial strains**	Heavy metal^*^								NaCl M (1 M = 58 g l^−1^) ^**^			
	**Cr mg l^−1^**				**Cd mg l^−1^**	**Cu mg l^−1^**	**Pb mg l^−1^**	**Zn mg l^−1^**				
	**500**	**1000**	**2000**	**3000**	**100**	**100**	**100**	**100**	**1.0 M**	**2.0 M**	**3.0 M**	**3.5 M**
*Staphylococcus saprophyticus* PJSI1	+	+	+	−	−	+	−	−	+	+	+	−
*Massilia* sp. PJSI12	+	+	−	−	−	−	−	+	+	−	−	−
*Ochrobactrum intermedium* PJSI13	+	+	−	−	−	+	+	+	+	−	−	−
*Arthrobacter* sp. PJRS17	+	−	−	−	−	−	+	+	+	−	−	−
*Pseudomonas aeruginosa* PJRS20	+	+	+	+	+	+	+	−	+	+	+	−
*Bacillus* sp. PJRS25	+	−	−	−	−	−	−	−	+	−	−	−
*Bacillus licheniformis* PJRI21	+	−	−	−	−	−	−	+	+	−	−	−
*Bacillus pumilus* PJSI41	+	−	−	−	+	+	−	+	+	−	−	−
*Staphylococcus* sp. PJSI46	+	+	−	−	−	+	+	+	+	−	−	−
*Aerococcus* sp. PJSI34	+	−	−	−	+	+	−	+	+	+	+	−
*Staphylococcus epidermidis* PJSI19	+	+	+	−	+	−	+	+	+	−	−	−
*Staphylococcus epidermidis* UK09	+	−	−	−	−	−	−	+	+	−	−	−
*Staphylococcus epidermidis* ASI	+	−	−	−	−	+	−	−	+	+	−	−
*Aerococcus* sp. PJSI36	+	−	−	−	+	−	−	+	+	+	−	−
*Pantoea stewartii* ASI11	+	+	+	+	+	+	+	+	+	+	−	−
*Ochrobactrum* sp. ASI14	+	+	+	−	+	−	+	−	+	−	−	−
*Bacillus aerophilus* SISI43	+	−	−	−	−	+	+	−	+	−	−	−
*Staphylococcus* sp. PJRI24	+	−	−	−	−	−	−	+	+	+	−	−
*Bacillus licheniformis* RSA27	+	−	−	−	−	−	−	−	+	−	−	−
*Microbacterium arborescens* HU33	+	+	+	+	+	+	+	+	+	+	+	−
*Aerococcusviridans* PASI10	+	+	−	−	−	−	+	−	+	−	−	−
*Brevundimonas vesicularis* PJSI37	+	+	+	−	−	+	−	−	+	−	−	−
*Enterobacter* sp. HU38	+	+	+	+	+	+	+	+	+	−	−	−
*Bacillus aquimaris* SISI39	+	+	+	−	−	−	−	+	+	−	−	−
*Pseudomonas* sp. PJRS31	+	+	+	−	−	−	−	+	+	−	−	−
*Pseudomonas stutzeri* RSAUK31	+	+	+	−	+	+	−	+	+	−	−	−

*+growth comparable to non-supplemented control plates within 24 h incubation*.

*−No growth even after 72 h incubation*.

* *Isolates were streaked on media containing varying heavy metal concentrations (measured as mg L^-*1*^ LB) in triplicates. Growth recorded in comparison to non-supplemented control plates*.

** *NaCl concentration in LB media (% w/v) at which growth was recorded*.

### Resistance to other heavy metals and NaCl

Most of the isolated rhizosphere and endophytic bacteria exhibited tolerance to different heavy metals (Cd, Cu, Pb, and Zn) and NaCl (Table 3). Three isolates exhibited tolerance to all the tested heavy metals (100 mg l^−1^). Strain ASI11 (99% 16S rRNA gene identity to *P. stewartii)*, strain HU33 (99% 16S rRNA gene identity to *M. arborescens*) and strain HU38 (99% 16S rRNA gene identity to *Enterobactor* sp.) showed maximum (300 mg l^−1^) resistance toward As, Cd, Pb, and Zn (data not shown). The growth of the isolates in the presence of NaCl was also evaluated. All the isolated bacteria were able to grow at 1 M (58 g l^−1^) NaCl, only four isolates showed resistance to higher concentration of NaCl, i.e., 3 M (174 g l^−1^), and none of them showed tolerance to 3.5 M (203 g l^−1^) NaCl.

### Plant growth-promoting activities

Most of the isolated strains exhibited one or more plant growth-promoting activities (Table 4). Only four shoot endophytes did not exhibit any tested plant growth-promoting activity. Eighteen strains exhibited ACC deaminase activity, 10 showed phosphorous solubilization activity, 7 showed IAA production potential and 11 were able to produce siderophores. Three isolates (*P. stewartii* strain ASI11, *M. arborescens* strain HU33 and *Enterobacter* sp. strain HU38) which exhibited all four tested plant growth-promoting activities as well as tolerance to higher levels of heavy metals and salt were selected for further analysis.

**Table 4:** **Plant growth promoting activities of bacteria isolated from the rhizosphere, root interior and shoot interior of *Prosopis juliflora* growing on the tannery effluent contaminated soil**.

**Bacterial strains**	**ACC-deaminase**	**P-solubilization**	**IAA production**	**Siderophore production**
*Staphylococcus saprophyticus* PJSI1	**+**	**+**	**−**	**−**
*Massilia* sp. PJSI12	**+**	**−**	**−**	**−**
*Ochrobactrum intermedium* PJSI13	**+**	**−**	**−**	**+**
*Arthrobacter* sp. PJRS17	**+**	**+**	**−**	**+**
*Pseudomonas aeruginosa* PJRS20	**+**	**−**	**−**	**+**
*Bacillus* sp. PJRS25	**+**	**−**	**−**	**−**
*Bacillus licheniformis* PJRI21	**+**	**−**	**−**	**−**
*Bacillus pumilus* PJSI41	**+**	**−**	**−**	**−**
*Staphylococcus* sp. PJSI46	**−**	**+**	**−**	**+**
*Aerococcus* sp. PJSI34	**+**	**−**	**−**	**−**
*Staphylococcus epidermidis* PJSI19	**+**	**−**	**+**	**+**
*Staphylococcus epidermidis* UK09	**+**	**−**	**−**	**−**
*Staphylococcus epidermidis* ASI	**−**	**−**	**−**	**−**
*Aerococcus* sp. PJSI36	**−**	**−**	**−**	**−**
*Pantoea stewartii* ASI11	**+**	**+**	**+**	**+**
*Ochrobactrum* sp. ASI14	**+**	**+**	**−**	**+**
*Bacillus aerophilus* SISI43	**−**	**−**	**+**	**+**
*Staphylococcus* sp. PJRI24	**−**	**−**	**−**	**−**
*Bacillus licheniformis* RSA27	**−**	**+**	**−**	**−**
*Microbacterium arborescens* HU33	**+**	**+**	**+**	**+**
*Aerococcus viridans* PASI10	**+**	**+**	**+**	**+**
*Brevundimonas vesicularis* PJSI37	**−**	**−**	**+**	**−**
*Enterobacter* sp. HU38	**+**	**+**	**+**	**+**
*Bacillus aquimaris* SISI39	**+**	**+**	**−**	**−**
*Pseudomonas* sp. PJRS31	**+**	**−**	**−**	**−**
*Pseudomonas stutzeri* RSAUK31	**−**	**−**	**−**	**−**

### Effect of bacterial inoculation on plant growth and phytoremediation efficacy

**Figure 1:**
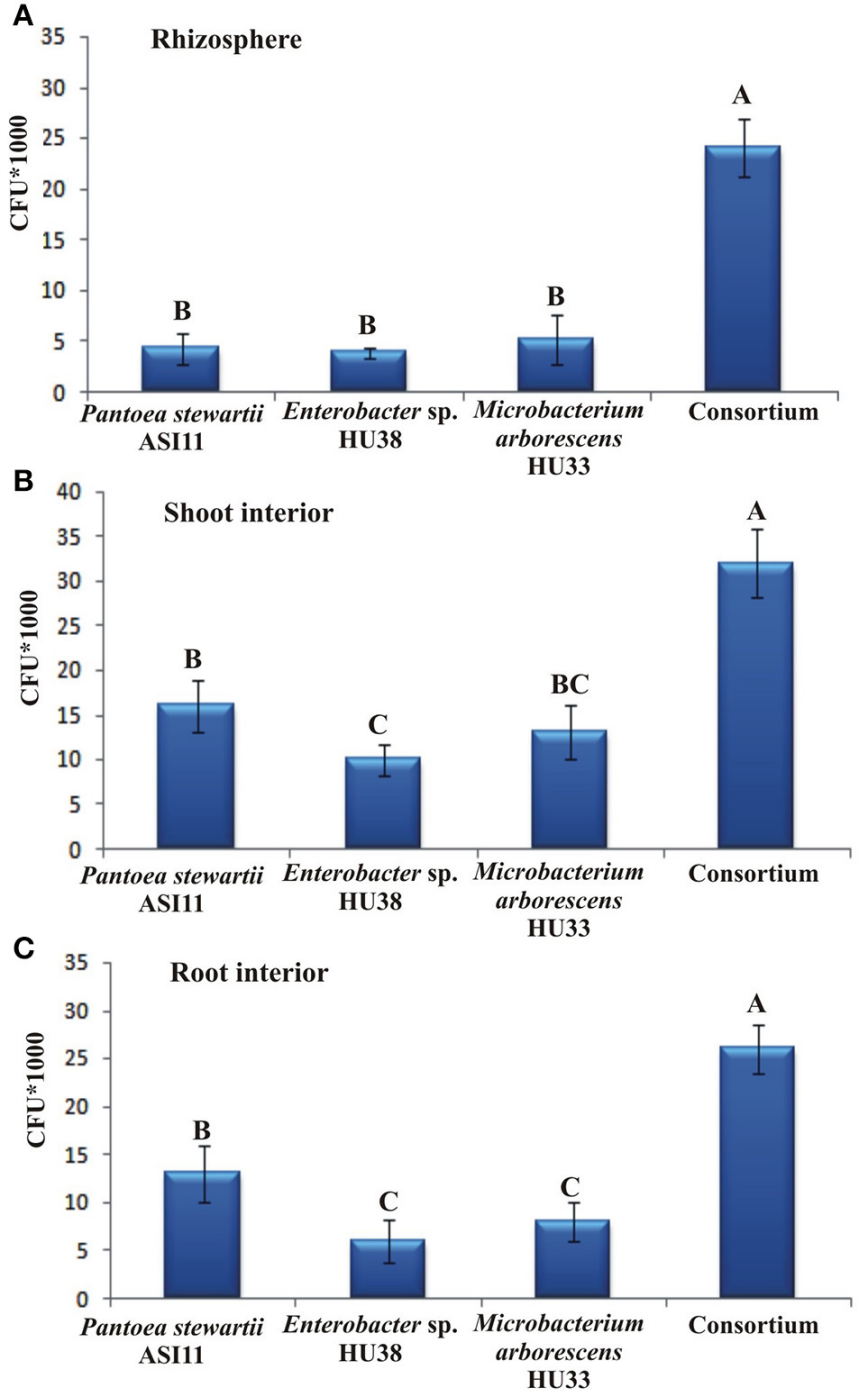
**Persistance of the inoculated bacteria in the rhizosphere (A), shoot interior (B) and toot interior (C) of ryegrass vegetated on the tannery effluent contaminated soil**.

The effect of the isolated endophytic bacteria on growth of ryegrass vegetated in the tannery effluent contaminated soil was evaluated in a pot experiment. Comparatively less seed germination, shoot and root length and weight were obtained by the plants vegetated in the tannery effluent contaminated soil than the plants vegetated in agricultural soil (Table 5). Generally, bacterial inoculation improved seed germination, root and shoot length and weight. However, the application of a combination of three strains was found more efficient as compared to single-strain inoculum. Moreover, bacterial inoculation enhanced the accumulation of Cr in the root and shoot of ryegrass (Table 6). Maximum Cr accumulation was observed in the root and shoot of the plants inoculated with the multi-strain inoculation. The inoculated bacteria showed better persistence in the root and shoot than in the rhizosphere and maximum persistence was observed when the strains were applied in combination (Figure 1).

**Table 5:** **Effect of bacterial inoculation on seed germination, root and shoot length and dry weight of ryegrass vegetated on the tannery effluent contaminated soil**.

**Treatment**	**Seed germination**	**Root**		**Shoot**	
	**(%)**	**Length (cm)**	**Weight (g)**	**Length (cm)**	**Weight (g)**
Control	80 (3.5)^a^	29.6 (1.2)^a^	61 (3.8)^a^	65 (4.3)^a^	29 (1.4)^a^
Contaminated soil	53 (2.2)^d^	15.6 (1.6)^d^	31 (2.7)^d^	34 (3.5)^d^	15 (0.7)^d^
*Pantoea stewartii* ASI11	62 (2.6)^c^	20.5 (0.9)^c^	37 (3.2)^cd^	40 (2.8)^c^	21 (1.1)^c^
*Enterobacter* sp. HU38	65 (2.8)^c^	19.4 (1.1)^c^	41 (2.9)^c^	41 (3.4)^c^	19 (0.8)^c^
*Microbacterium arborescens* HU33	64 (2.5)^c^	21.8 (0.8)^c^	40 (1.5)^c^	39 (2.3)^c^	20 (1.2)^c^
Consortium^*^	72 (3.1)^b^	26.84 (1.4)^b^	47 (3.6)^b^	53 (3.8)^b^	24 (1.8)^b^

*Each value is the mean of three replicates, means in the same column followed by the same letter (a, b, c, d) are not significantly different at a 5% level of significance, the standard error of three replicates is presented in parentheses*.

* *Mixture of Pantoea stewartii ASI11, Microbacterium arborescens HU38, and Enterobacter sp. HU33*.

**Table 6:** **Effect of bacterial inoculation on the accumulation of Cr in the root and shoot of ryegrass vegetated on the tannery effluent contaminated soil**.

**Treatment**	**Soil (Cr mg kg^−1^)**	**Root (Cr mg kg^−1^)**	**Shoot Cr mg kg^−1^)**
Ryegrass	1923 (58)^a^	176 (8)^c^	114 (6)^c^
*Pantoea stewartii* ASI11	1552 (46)^b^	435 (10)^b^	240 (9)^b^
*Enterobacter* sp. HU38	1605 (34)^b^	384 (12)^b^	213 (8)^b^
*Microbacterium arborescens* HU33	1596 (62)^b^	380 (16)^b^	222 (7)^b^
Consortium^*^	1250 (37)^c^	598 (13)^a^	356 (5)^a^

*Each value is the mean of three replicates, means in the same column followed by the same letter (a, b, c, d) are not significantly different at a 5% level of significance, the standard error of three replicates is presented in parentheses*.

* ^*^Mixture of Pantoea stewartii ASI11, Microbacterium arborescens HU38, and Enterobacter sp. HU33.

## Discussion

Although plants need some heavy metals as essential micronutrients, their excess in soil inhibits plant growth. The heavy metal tolerating capacity of plants mainly depends on plant species or genotype and the concentration of specific heavy metals in the environment (Pulford and Watson, 2003; Jamal, 2006; Leitenmaier and Küpper, 2013). There are no standards of heavy metals concentration in soil set by Pakistan. However, the limit of soil Cr in agricultural land, residential area, and commercial-industrial area were 500, 600, and 800 mg kg^−1^ in Germany and 750, 250, and 800 mg kg^−1^ in Canada, respectively (Balasoiu et al., 2001). In Canada, the allowed concentration of Cd, Ni, Pb and Zn in agricultural soil are 3, 50, 150, 200, and 600 mg kg^−1^ soil. In this study, high concentration of Cr and other heavy metals was observed in the rhizosphere, root, and shoot of *P. juliflora* growing on the tannery effluent contaminated soil (Table 1). Several earlier studies also reported that this plant can accumulate high concentration of different metals in its roots and shoots (Rai et al., 2004; Senthilkumar et al., 2005). This might be one of the reasons that *P. juliflora* hosts several bacteria in its rhizosphere and endosphere which can tolerate high concentration of heavy metals.

In the present study, a higher richness of culturable Cr-resistant bacteria were found in the endosphere than in the rhizosphere and the predominant genera were *Bacillus*, *Staphylococcus* and *Aerococcus*. This might be due to better nutrients and environmental conditions inside the plant tissues than in the soil (Compant et al., 2010; Afzal et al., 2013), but it may be also due to better culturability of endophytes. Another possible reason could be higher concentrations of different toxic heavy metals in the soil than within plant tissues. Several studies showed that metals influence microorganisms by adversely affecting their growth, morphology and biochemical activities, resulting in a decrease in their biomass and numbers (Giller et al., 1998; Abou-Shanab et al., 2005). Mostly, endophytes were different from rhizosphere strains and also roots and shoots hosted distinct taxa. Only few strains were isolated from the rhizosphere of *P. juliflora*, such as strain PJRS17 (99% 16S rRNA gene identity to *Arthrobacter* sp.), strain PJRS20 (99%16S rRNA gene identity to *Pseudomonas aeruginosa*), strain PJRI21 (99% 16S rRNA gene identity to *Bacillus licheniformis*), strain RSA27 (99% 16S rRNA gene identity to *Bacillus licheniformis*) and strain RSAUK31 (99% 16S rRNA gene identity to *Pseudomonas stutzeri*) as shown in Table 2. It has been reported that most endophytes originate from the rhizosphere (Sessitsch et al., 2002; Compant et al., 2010), however, the plant apoplast offers different growth conditions and therefore different strains efficiently colonize the plant interior.

Tolerance of the isolates toward Cr was the first parameter evaluated, and all isolates were able to tolerate this metal up to 500 mg l^−1^. Cr, together with Cd, Cu, Pb, and Zn are the main contaminants in the tannery effluent contaminated soil (Khan, 2001; Tariq et al., 2009; Afzal et al., 2014a). Among the isolates, four strains showed resistance to very high concentration of Cr (3000 mg l^−1^) which include *Pseudomonas aeruginosa* sp. strain PJRS20, *Pantoea stewartii* sp. strain ASI11, *Microbacterium arborescens* sp. strain HU33 and *Enterobacter* sp. strain HU38. In many other studies Cr-resistant bacteria were also isolated, however, they exhibited resistance to comparatively lower concentration of this metal (Srinath et al., 2002; Viti et al., 2003; Chatterjee et al., 2009). The Cr-resistant isolates can also tolerate other heavy metals and NaCl (174 g l^−1^), suggesting the potential use of these bacteria in the bacterial-assisted phytoremediation of soil contaminated with heavy metals and also for restoration of the saline soil.

Most of the isolated bacteria also exhibited one or more plant growth-promoting activities. It could be one of the possible reasons of the survival and growth of *P. juliflora* in the highly contaminated soil. Among the isolates, 69% exhibited ACC deaminase activity, which is an important function of plant growth-promoting bacteria, it causes the reduction of stress ethylene in plants (Glick, 2010; Glick and Stearns, 2011; Sessitsch et al., 2013). Comparatively, a low number of isolated strains showed IAA production (27%), P-solubilization (35%) and siderophore production (42%). IAA production enhances the root surface area and nutrients uptake by plants (Shagol et al., 2014). The phosphate-solubilizing activity can enhance the availability of phosphorous and heavy metals to the plants (Fitz and Wenzel, 2002). Three strains (*P. stewartii* strain ASI11, *M. arborescens* strain HU33 and *Enterobacter* sp. strain HU38) exhibiting multiple plant growth promoting potential as well as tolerance to higher levels of heavy metals and salt were selected for use *in vitro* plant-inoculation assay. Altogether, plant growth-promoting bacteria have the potential to improve plant growth and may either increase uptake of heavy metals by plants or stabilize heavy metals in soils preventing further uptake (Glick, 2010; Rajkumar et al., 2012; Sessitsch et al., 2013).

In this study, comparatively less seed germination and root and shoot development was observed with plants cultivated in the tannery effluent contaminated soil as compared to the plants vegetated in control agricultural soil (Table 5). The presence of Cr and other heavy metals in soil reduces seed germination and plant growth (Khan, 2001; Sagar et al., 2012; Carvalho et al., 2013; Lin et al., 2014). Despite the toxic effects of heavy metals present in the contaminated soil, inoculation of Cr-resistant plant growth-promoting bacteria increased seed germination and root and shoot development. Particularly, bacterial consortium comprising three individual strains improved plant growth and development to a greater extent than single strain application. Similarly, it was found that bacterial inoculation reduced Cr toxicity and improved seed germination and plant growth (Chatterjee et al., 2009). In this study, the ability of the inoculated endophytic bacteria to improve plant growth in the heavy metal contaminated soil might be due to the combined effect of heavy metal tolerance ability as well as plant growth-promoting activities (Glick, 2010).

Bacterial populations of inoculated strains in the rhizosphere and endosphere of ryegrass were determined and we found that the inoculated strains were able to persist in the rhizosphere and endosphere of the plant vegetated in the tannery effluent contaminated soil (Figure 1). Although Cr-resistant bacteria were applied on the soil surface, high numbers of bacterial cells were found within plant tissues. The plant interior might provide a more protective and less toxic environment than the rhizosphere (Afzal et al., 2012). Similarly in previous studies, applied endophytic bacteria exhibited higher levels of colonization and activity in the endosphere than the rhizosphere (Andria et al., 2009; Afzal et al., 2011; Yousaf et al., 2011).

In this study, ryegrass was found to be able to remove Cr and other heavy metals from the tannery effluent contaminated soil. The application of Cr-resistant plant growth-promoting endophytic bacteria to ryegrass further enhanced the removal of Cr (Table 6) and other heavy metals (data not shown) from the soil. The maximum accumulation of heavy metals was found in the roots and shoots of the plants inoculated with the combination of three bacterial strains. Similarly, earlier studies reported that the inoculation of Cr-resistant plant growth-promoting bacteria enhanced the heavy metals uptake by plants (Faisal and Hasnain, 2006; Rajkumar et al., 2006; Wani et al., 2008; Arzanesh et al., 2009). Enhanced heavy metals translocation in plant tissues can be attributed to the IAA production and phosphate solubilization activity of the inoculated strains (Husen, 2013). The present study suggests that the use of Cr-resistant plant growth-promoting bacteria protects the plant against the inhibitory effects of heavy metals present in the tannery effluent contaminated soil and facilitates the transportation of heavy metals from soil into above ground plant biomass.

Plant–bacteria partnerships can be exploited to enhance phytoremediation efficiency of soil and water contaminated with organic and inorganic pollutants (Weyens et al., 2009; Khan et al., 2013; Afzal et al., 2014b). The beneficial effects of heavy metal-resistant and plant growth-promoting bacteria include reduced heavy metals toxicity and accelerated root development, resulting in better access to nutrients and water and thus faster initial growth, leading to enhanced remediation of contaminated soil and water and environmentally and economically sustainable plant biomass production. Improved yields on contaminated land might also reduce the need to clear and use additional areas of land for food, feed fiber and biofuel feedstock production for a growing world population, consequentially saving native ecosystems and biodiversity. Overall, the combined use of plants and bacteria can act as decontaminators by improving phytoremediation or protecting the food chain by decreasing the levels of pesticide residues in crops.

We have found that *P. juliflora* hosted 26 culturable Cr-resistant bacteria in its endosphere and rhizosphere and their inoculation to ryegrass improved plant growth and the remediation of tannery effluent contaminated soil, suggesting their potential use in the remediation of heavy metal contaminated soil. The stimulatory effects of Cr-resistant plant growth-promoting endophytic bacteria on ryegrass growth might be due to the additive effects of different plant growth-promoting properties of the isolated endophytic bacteria. Further plant-inoculation experiments (pair-wise in different combinations) are needed to better understand the stimulatory effects of the combined inoculation strategy. The very high level of metal tolerance of the isolated rhizosphere and endophytic bacteria of *P. juliflora* makes them interesting candidates for further studies on the genes involved in this tolerance. Plants growing on tannery effluent contaminated sites could be excellent ecosystems to isolate bacterial genes involved in metal resistance and/or plant growth promotion.

### Conflict of interest statement

The authors declare that the research was conducted in the absence of any commercial or financial relationships that could be construed as a potential conflict of interest.
